# The Effect of *Crocus sativus* L. and Its Constituents on Memory: Basic Studies and Clinical Applications

**DOI:** 10.1155/2015/926284

**Published:** 2015-02-01

**Authors:** Nikolaos Pitsikas

**Affiliations:** Department of Pharmacology, School of Medicine, Faculty of Health Sciences, University of Thessaly, Panepistimiou 3, Biopolis, 41500 Larissa, Greece

## Abstract

Memory-related disorders are a common public health issue. Memory impairment is frequent in degenerative diseases (such as Alzheimer's disease and Parkinson disease), cerebral injuries, and schizophrenia. The dried stigma of the plant *Crocus sativus* L. (*C. sativus*), commonly known as saffron, is used in folk medicine for various purposes. Several lines of evidence suggest that *C. sativus* and its constituents are implicated in cognition. Here we critically review advances in research of these emerging molecular targets for the treatment of memory disorders, and discuss their advantages over currently used cognitive enhancers as well remaining challenges. Current analysis has shown that *C. sativus* and its components might be a promising target for cognition impairments.

## 1. Introduction

*Crocus sativus* L. (*C. sativus*) is a perennial herb member of the Iridaceae family, the line of Liliaceae. This plant is cultivated in many countries such as Azerbaijan, China, France, Greece, Egypt, India, Iran, Israel, Italy, Mexico, Morocco, Spain, and Turkey. Its product is the well-known spice called saffron. Saffron, in filaments, is the dried dark-red stigmas of* C. sativus* flower [1]. One stigma of saffron weighs about 2 mg and each flower has three stigmata; 150000 flowers must be carefully picked one by one to obtain 1 kg of spice. Saffron has a distinct colour, flavour, and odour. It is used both as a spice for flavouring and colouring food preparations and as a perfume. The stigmas of it are also used in folk medicine as an anticatarrhal, eupeptic, antispasmodic, expectorant, emmenagogue, and nerve sedative (for review see [2]). Interestingly, saffron has widely been used in the Persian traditional medicine for memory problems [3].

## 2. Chemistry of* C. sativus*

Chemical analysis of* C. sativus* stigmas has shown the presence of about 150 volatile and nonvolatile compounds; fewer than 50 constituents, however, have been identified so far [4]. The volatiles consist of more than 34 components that are terpenes, terpene alcohols, and their esters among which safranal is the main component. Nonvolatile compounds comprise crocins, crocetin, picrocrocin safranal, and flavonoids (quercetin and kaempferol) [5].

Particularly, crocins, glucosyl esters of crocetin, are water-soluble carotenoids and are responsible for saffron's characteristic colour. Picrocrocin, glycoside of safranal, is responsible for the bitter taste of the spice and is precursor of safranal. Safranal, the main component of the distilled essential oil, is a monoterpene aldehyde, responsible for its characteristic aroma [6, 7].

## 3. Pharmacological Actions of* C. sativus* and Its Constituents

### 3.1. Safety Studies

Toxicity studies have demonstrated that the hematological and biochemical parameters were within normal range in mice treated with saffron extracts [8]. It has also been reported that the oral LD_50_ of saffron was 20.7 g/kg administered as decoction in mice [9]. Further, a recent work investigated either the acute (up to 3 g, both orally (p.o.) and intraperitoneally (i.p.)) or the subchronic effects of crocin (15–180 mg/kg, i.p.) in different biochemical, hematological, and pathological parameters in rodents. The results of this study evidenced that at pharmacological doses crocin did not display appreciable toxicity [10].

Interestingly, the findings of clinical studies suggest that both* C. sativus* extracts and crocin display a relative safe and normal pharmacological profile. Specifically, in a double-blind, placebo-controlled trial conducted on healthy volunteers a one-week treatment with saffron (200–400 mg/day) did not evidence particular alterations [11]. Moreover, the results of another double-blind, placebo-controlled study performed in healthy volunteers also showed that administration for one month of crocin (20 mg/day) did not elicit significant alterations of different hematological, biochemical, hormonal, and urinary parameters recorded [12].

### 3.2. Effects of* C. sativus* and Its Constituents on Cancer Therapy, Hepatotoxicity, Nociception, Inflammation, and Cardiovascular System

Preclinical pharmacological studies have demonstrated that* C. sativus* crude extracts and purified chemicals possess antitumour effects (e.g., [8, 13, 14]). Saffron and its ingredients display antinociceptive and anti-inflammatory properties [15], reduce atherosclerosis [16] and hepatic damage [17], counteract hyperlipidaemia [18], protect from myocardial injury [19], and display antihypertensive action [20, 21]. The effects exerted by saffron and its components in the aforementioned pathologies were also extensively discussed in different reviews (e.g., [2, 22–24]).

The outcome of these preclinical studies indicates that* C. sativus* and its components exert a beneficial action in several pathologies. It is important to underline, however, that up to our days there is no clinical information on the potential efficacy of saffron and its constituents in the aforementioned pathologies. Future research aiming to prove the efficacy of this spice observed in preclinical experimentations in humans is required.

### 3.3. Effects of* C. sativus* and Its Constituents on Central Nervous System (CNS)

#### 3.3.1. Affective Disorders

Experimental evidence indicated that crocins displayed an anxiolytic-like effect in procedures assessing anxiety and obsessive compulsive disorder (OCD) in rats [25, 26]. Interestingly, a study carried out in mice suggested that the aqueous extracts of* C. sativus* and safranal but not crocin have anxiolytic and hypnotic effects [27].

*C. sativus* and its active components crocin and safranal have shown antidepressant-like effects in animal models of depression [28]. Importantly, clinical research reinforced preclinical results and proposed that saffron is efficacious for the treatment of mild-to-moderate depression [29, 30]. In this context, it has been shown that saffron antagonized sexual dysfunction induced by the selective serotonin reuptake inhibitor fluoxetine in humans [31, 32].

Recently, it has been reported that crocins were found effective in antagonizing psychotomimetic effects in rats related to the hypofunction of the glutamatergic system. These results suggest that further studies should be carried out aiming to elucidate whether or not these active components of saffron might constitute a potential candidate for the treatment of schizophrenia [33].

#### 3.3.2. Anticonvulsant Activity, Neurodegeneration

Studies performed in rodents revealed a certain anticonvulsant activity of aqueous and ethanolic extracts of* C. sativus* and its active component safranal [34, 35]. Saffron and its active constituents affect a number of different neural processes, for example, conferred neuroprotection in a rat model of Parkinson disease (PD) [36]. In addition,* in vitro* and* in vivo* preclinical experimentations propose a protective role for saffron extracts, crocin and safranal, in cerebral ischemia [37–40].

## 4. Effects of* C. sativus* and Its Constituents on Cognition

Dementia is one of the major medical illnesses at older age, where the patient's language, attention, and memory are compromised. Amnesia is among the typical features of dementia and is characterized by the inability to form memories or total or partial loss of memory secondary to cerebral malfunction following degenerative diseases such as Alzheimer's disease (AD), cerebral infections such as herpes or encephalitis, traumatic injuries such as stroke, alcohol, or drug abuse, emotional events such as psychological trauma, and affective disorders (schizophrenia).

AD is the most common form of dementia. The prevalence of AD is strongly correlated with increasing age and is a consequence of progressive neurodegeneration occurring over a period of several years. The neurodegeneration leads to a gradual decline in cognitive, functional, and behavioural processes, producing characteristic symptoms such as memory loss, confusion agitation, and difficulties performing the activities of daily living [41].

No conventional or alternative therapy is currently available to cure amnesia. Current therapeutic strategies for amnesia are mainly focused on enhancing or restoring cerebral circulation, restoring the cholinergic neurotransmission and scavenging free radicals. In the management of amnesia, as well as AD, sustained treatment with acetylcholinesterase (AChE) inhibitors including donepezil, rivastigmine, galantamine, tacrine, and the N-methyl-D-aspartate (NMDA) receptor antagonist memantine has been used [42, 43]. However, these drugs have modest efficacy and induce severe side effects [44, 45].

### 4.1. Preclinical Studies

An overview of the preclinical literature regarding the effects of* C. sativus* and its constituents on memory is provided in Table 1. Oral administration of extracts of* C. sativus* (125–500 mg/kg) reversed ethanol but not scopolamine-induced memory impairments in the passive avoidance paradigm in mice [46]. Similarly, administration of the active constituent of* C. sativus* crocin (50–200 mg/kg, p.o.) but not picrocrocin counteracted ethanol-induced performance deficits in the mice in the same memory test [47]. In addition, a single intraperitoneal injection of* C. sativus* extracts (30–60 mg/kg) antagonized scopolamine-induced performance deficits in the passive avoidance task and extinction of recognition memory in rats [48].

The active components of saffron crocins (15–30 mg/kg, i.p.) counteracted scopolamine-induced performance deficits in the novel object recognition test (NORT) and in the radial water maze task in rats. NORT and radial water maze task are procedures assessing recognition and spatial memory, respectively, in rodents [50]. The findings of the latter two studies [48, 50] clearly support a functional interaction between* C. sativus* and crocins with the cholinergic system. Moreover, chronic application of crocin (15–30 mg/kg, i.p.) attenuated performance deficits produced by administration of streptozotocin (STZ) in the passive avoidance and the Y-maze procedures in rats [51].

Chronic treatment with* C. sativus* extracts (60 mg/kg, i.p.) reversed age-related memory deficits in the 20-month-old mouse evidenced in the passive avoidance paradigm [52]. In a procedure evaluating spatial memory in rodents (Morris water maze) it was found that a 21-day treatment with* C. sativus* extracts (30 mg/kg, i.p.) or crocin (15–30 mg/kg, i.p.) reversed spatial learning and memory deficits produced by chronic stress in rats [53].

Chronic administration of* C. sativus* extracts (100–250 mg/kg, i.p.) and crocin (5–30 mg/kg, i.p.) was able to counteract spatial memory deficits in a model of cerebral ischemia in rats [38]. Using the same procedure and a chronic regimen of administration of crocin (100 mg/kg, p.o.), Naghizadeh et al. showed a protective effect of this constituent of* C. sativus* on spatial memory deficits and oxidative stress produced by STZ in rats [54]. Conversely, a study aiming to investigate potential neuroprotective effect of* C. sativus* extracts (60 mg/kg, i.p. chronically) against established aluminium (AlCl_3_) toxicity failed to attenuate the AlCl_3_-induced memory deficits in the passive avoidance task in mice. Reportedly, in this context, saffron treatment was found to attenuate different biochemical markers of brain functions which were altered by the AlCl_3_-induced toxicity [55].

Finally, a single injection of crocins (15–30 mg/kg, i.p.) reversed recognition memory deficits produced by the NMDA receptor antagonist ketamine in rats eliciting thus the implication of this active constituent of* C. sativus* in schizophrenia-related cognitive deficits. These findings also propose a functional interaction between crocins and the glutamatergic system [33]. In this context, it has been reported that acute systemic administration of safranal reduced kainic acid-induced increase of extracellular glutamate concentrations in the rat hippocampus [59] and* C. sativus* extracts inhibited glutamatergic synaptic transmission in rat cortical brain slices [60]. Collectively, these results suggest that this reduction of glutamate levels by saffron and its constituents might be critical for the beneficial action exerted by crocins on ketamine-induced behavioural deficits.

### 4.2. Clinical Studies

An overview of the clinical literature regarding the effects of* C. sativus* on cognition is provided in Table 2. Up to now, few trials were carried out aiming to assess the effects of saffron in humans suffering from memory disorders.

A first study was carried out in 46 patients with mild-to-moderate AD. Patients received saffron (30 mg/day, p.o.) or placebo for 16 weeks. The results of this randomized, double-blind study suggested that saffron might be both safe and effective in mild-to-moderate AD patients [56].

A subsequent clinical trial of the same group of scientists was designed and performed aiming to evaluate in a larger number of patients (55 patients participated) suffering from mild-to-moderate AD the potential effects of* C. sativus* on cognition. In this 22-week, multicentre, double-blind controlled trial participants randomly were assigned to receive saffron (30 mg/day, p.o.) or the reference compound, the AChE inhibitor donepezil (10 mg/day, p.o.). The findings of this phase II study provided preliminary evidence of a possible therapeutic effect of* C. sativus* in AD. Specifically, saffron's efficacy was not different than that of donepezil and some adverse effects that occurred after donepezil treatment (vomiting) were absent in the saffron-treated patients [57].

Finally, a recent clinical work compared the efficacy and safety of* C. sativus* with the NMDA receptor antagonist memantine in patients suffering from moderate-to-severe AD. In this one-year, double-blind clinical trial participated 68 patients which were randomly assigned to receive saffron (30 mg/day, p.o.) or memantine (20 mg/day, p.o.). The results of this study did not reveal any difference either in terms of efficacy or in terms of safety between patients that received either memantine or saffron [58].

## 5. Mechanism of Action of* C. sativus* and Its Constituents

The mechanism(s) underlying effects of saffron and its active constituents on cognition is still a matter of investigation. Among the potential mechanisms, the promotion of long-term potentiation (LTP), the antiamyloidogenic activity, its inhibitory action on the AChE activity, and their potent antioxidant properties are proposed to explain their action on cognition.

Specifically, LTP is an activity-dependent form of modified transmission efficacy at synapses [61]. This type of neuronal plasticity, first shown in the hippocampus, in the meanwhile, has been found to occur in various brain regions, thus being a widespread phenomenon in the CNS. In addition, learning and memory occur as a result of changes in the efficacy of synaptic transmission. Reportedly, in this context, it has been revealed that crocin promoted the hippocampal LTP [49].

Aggregation and deposition of amyloid-*β* (A*β*) peptides is the main molecular process underlying AD. It has been shown that saffron stigmas' extract interacts with these peptides and prevented A*β* fibrillogenesis and amyloid formation in an* in vitro* model of AD [62]. In addition, the results of* in vitro* enzymatic and molecular docking studies indicate that* C. sativus* and crocetin but not safranal inhibit AChE by binding in two different loci, the catalytic center and the peripheral anionic sites. This AChE inhibitory activity displayed by* C. sativus* and crocetin promotes an increase of the synaptic acetylcholine levels, a neurotransmitter critically involved in cognitive functions [63].

Consistent experimental evidence proposes a key role for oxidative stress in the pathogenesis of different neurodegenerative disorders including AD and PD and in pathological conditions such as brain injuries [64–67]. In addition, although the pathogenesis of schizophrenia remains unknown, a possible relationship between oxidative stress and the disease has also been proposed [68, 69]. In support of this view, it was reported that ketamine increased oxidative stress in the brain of rats [70]. Therefore, an alternative hypothesis to explain the beneficial action exerted by saffron and crocin in memory disorders is based on the well-known antioxidant properties of* C. sativus* and its constituents. In line with this, a wide variety of studies revealed the antioxidant properties of* C. sativus* [39, 52, 53, 55, 62], crocin [40, 53, 71–73], crocetin [52], and safranal [37].

## 6. Conclusions

Accumulating evidence indicates that* C. sativus* and its major component crocin are significantly involved in cognition. Preclinical studies demonstrated their efficacy in attenuating memory disorders in animal models related to AD, cerebral injuries, or schizophrenia.

Clinical research has evaluated the efficacy of saffron, but not of crocin, in a narrow range of memory disorders (AD). The results indicated that its effects on cognition, although modest, were not different than those expressed by donepezil and memantine. In this context, it is important to emphasize the good safety profile of saffron which was revealed in all clinical experimentations. There is no information, however, on the potential efficacy of saffron and crocin in memory disorders which occurred in other pathologies such as brain ischemia, traumatic brain injury, and cognitive deficits related to schizophrenia. The potential role of saffron and crocin as adjunctive agents, in combination with an AChE inhibitor or memantine, for the treatment of memory disorders has not been investigated so far. Future research should address these issues.

## Figures and Tables

**Table 1:** Effects of* Crocus sativus* L. and its constituents on cognition. Preclinical studies.

Species	Agent	Dose range	Route	Memory task	Effect	Reference
Mouse	CsE Ethanol Scopolamine	125–250–500 mg/kg 30%, 10 mL/kg 0.5 mg/kg	p.o. acute p.o. acute i.p. acute	Passive avoidance	Reversed ethanol but not scopolamine-induced deficits	[46]
Mouse	Crocin Picrocrocin Ethanol	50–100–200 mg/kg 50–100–200 mg/kg 30–40%, 10 mL/kg	p.o. acute p.o. acute p.o. acute	Passive avoidance	Crocin but not picrocrocin reversed ethanol-induced deficits	[49]
Rat	CsE Scopolamine	10–30–60 mg/kg 0.75 mg/kg	i.p. acute s.c. acute	Novel object recognition Passive avoidance	Reversed (30–60 mg/kg) scopolamine-induced and delay-dependent memory deficits	[48]
Rat	Crocins Scopolamine	15–30 mg/kg 0.2 mg/kg	i.p. s.c..	Novel object recognition Radial water maze	Given acutely reversed scopolamine-induced recognition memory deficits, chronic treatment (5 days) counteracted scopolamine-induced performance deficits in the radial water maze task	[50]
Rat	Crocin Streptozotocin	15–30 mg/kg 3 mg/kg	i.p. chronic i.c.v. acute	Y-maze Passive avoidance	Chronic treatment (30 mg/kg, for three weeks) reversed streptozotocin-induced performance deficits in the Y-maze and passive avoidance task	[51]
Aged mouse	CsE	60 mg/kg	i.p. chronic	Passive avoidance	Chronic treatment (7 days) reversed age-related memory deficits	[52]
Rat	CsE Crocin	30 mg/kg 15–30 mg/kg	i.p. chronic i.p. chronic	Morris water maze	Chronic treatment (21 days) reversed chronic stress-induced spatial memory deficits	[53]
Rat	CsE Crocin	50–100–250 mg/kg 5–10–25 mg/kg	i.p. chronic i.p. chronic	Morris water maze	Chronic treatment (7 days) of CsE (100–250 mg/kg) and crocin reversed cerebral ischemia-induced spatial memory deficits	[38]
Rat	Crocin Streptozotocin	100 mg/kg 3 mg/kg	p.o. chronic i.c.v. (2 infusions)	Morris water maze	Chronic treatment (21 days) reversed streptozotocin- induced spatial memory deficits	[54]
Mouse	CsE Aluminium (AlCl_3_)	60 mg/kg 50 mg/kg	i.p. chronic p.o. chronic	Passive avoidance	Ineffective to reverse AlCl_3_-induced memory deficits	[55]
Rat	Crocins Ketamine	15–30 mg/kg 3 mg/kg	i.p. acute i.p. acute	Novel object recognition	Reversed ketamine-induced recognition memory deficits	[33]

CsE, *Crocus sativus *extracts; i.c.v., intracerebroventricularly; i.p., intraperitoneally; p.o., orally; s.c., subcutaneously.

**Table 2:** Summary of clinical trials on *Crocus sativus* L., as treatment for AD.

Design of study	Duration of study	Severity of disease	Agent	Dose range	Route	Effect	Reference
Double-blind	16 weeks	Mild-to-moderate AD	*C. sativus *	30 mg/day	p.o.	Safe and with certain efficacy	[56]
Double-blind	22 weeks	Mild-to-moderate AD	*C. sativus * Donepezil	30 mg/day 10 mg/day	p.o. p.o.	Similar efficacy and better safety profile compared to donepezil	[57]
Double-blind	12 weeks	Moderate-to-severe AD	*C. sativus* Memantine	30 mg/day 20 mg/day	p.o. p.o.	Similar efficacy and safety profile compared to memantine	[58]

AD, Alzheimer's disease; *C. sativus*, *Crocus sativus* L.; p.o., orally.
